# DNAJB9 Is a Reliable Immunohistochemical Marker of Fibrillary Glomerulonephritis: Evaluation of Diagnostic Efficacy in a Large Series of Kidney Biopsies

**DOI:** 10.3390/biomedicines10092102

**Published:** 2022-08-27

**Authors:** Alessandro Gambella, Chiara Pitino, Antonella Barreca, Alberto Nocifora, Manuela Maria Giarin, Luca Bertero, Luigi Biancone, Dario Roccatello, Mauro Papotti, Paola Cassoni

**Affiliations:** 1 Pathology Unit, Department of Medical Sciences, University of Turin, 10126 Turin, Italy; 2 Pathology Unit, “Città della Salute e della Scienza di Torino” University Hospital, Via Santena 7, 10126 Turin, Italy; 3 Division of Nephrology Dialysis and Transplantation, “Città della Salute e della Scienza di Torino” University Hospital, Department of Medical Sciences, University of Turin, 10126 Turin, Italy; 4 CMID, Coordinating Center of the Network for Rare Diseases of Piedmont and Aosta Valley, Nephrology and Dialysis Unit (ERK-Net Member), San Giovanni Bosco Hub Hospital, University of Turin, 10144 Turin, Italy; 5 Pathology Unit, Department of Oncology, University of Turin, 10126 Turin, Italy

**Keywords:** fibrillary glomerulonephritis, DNAJB9, fibrils, electron microscopy, immunofluorescence, immunohistochemistry, nephrology, nephropathology

## Abstract

Fibrillary glomerulonephritis (FGN) is a rare glomerular disease characterized by a challenging diagnostic workup requiring ultrastructural identification of 20 nm-thick randomly oriented fibrillar deposits. However, the recent introduction of DNAJB9 as a putative diagnostic marker of FGN could thoroughly improve this diagnostic scenario. This study aims to assess the DNAJB9 immunohistochemical expression in a large series of FGN cases and to eventually confirm its role as a diagnostic marker of FGN. We evaluated the immunohistochemical expression of DNAJB9 (Rabbit Polyclonal, ThermoFisher) in a series of 77 FGN and 128 non-FGN cases diagnosed between January 1992 and June 2022 at the Pathology Unit of the AOU Città della Salute e della Scienza Hospital. DNAJB9 was expressed in 73 of the 74 evaluable FGN cases, mostly showing a strong glomerular positivity (68 cases). Additionally, DNAJB9 resulted positive in all challenging scenarios [early-stage (6), congophilic (4), combined (4), and uncertain (4) cases of FGN)]. DNAJB9 was negative in all non-FGN cases, eventually resulting in a specificity of 100% and sensitivity of 99%. In conclusion, we confirmed the role of DNAJB9 as a diagnostic marker of FGN. Its adoption in the clinical routine will allow a faster, more feasible, and more accurate FGN diagnosis.

## 1. Introduction

Kidney diseases with structured glomerular deposits represent a group of rare conditions with unspecific clinical presentation and a challenging diagnostic workup, requiring dedicated facilities (immunofluorescence and electron microscopy assessment), nephropathological expertise, and prolonged turn-around time even in tertiary referral centers. Indeed, as several conditions with different pathogenesis, prognosis, and treatment are included in this group (e.g., amyloidosis, cryoglobulinemic glomerulonephritis, lupus nephritis, fibronectin glomerulopathy, and immunotactoid glomerulonephritis), the identification and validation of specific and sensitive diagnostic markers are essential to timely identify and characterize the different diseases and appropriately guide the subsequent clinical management. 

In this setting, fibrillary glomerulopathy (FGN) is a rare primary glomerular disease representing less than 1% of native kidney diagnoses, mainly affecting the adult population with a slight female predominance [1]. FGN was first described in 1977 but its specific etiology has not been clearly identified, whereas a putative association with autoimmune diseases, non-hematological neoplasia, and HCV infection has increasingly emerged [2]. From a clinical perspective, FGN patients usually present proteinuria (almost 40% nephrotic) and hematuria; since no specific treatment has been identified, FGN harbors an overall poor prognosis with a progressive evolution to chronic end-stage kidney disease within 2–4 years from diagnosis [1,3]. FGN diagnostic workup is complex and challenging, as clinical and laboratory findings are not specific, thus requiring an extensive histopathological characterization [4]. Indeed, light microscopy (LM) is variable and aspecific, ranging from subtle to unremarkable alterations in the early stage to diffuse mesangial sclerosis or endocapillary proliferation and crescentic [1,3,5,6,7]. The immunofluorescence (IF) profile is characterized by IgG and C3 intra-glomerular positivity (with a variable IgG1 and IgG4 predominance) and the staining pattern is usually very typical with an irregular flocculent positivity, but in some cases a band-like staining is observed [1,3,5,6]. Of note, the most significant diagnostic feature is represented by the ultrastructural identification of randomly oriented, straight, and non-branching fibrils [6]. Additionally, FGN fibrils features are characterized by the lack of a hollow center, a diameter range between 10 and 30 nm (mean of 20 nm), and the mesangial location, but they could be observed in the glomerular basement membrane (GBM), vascular walls, or interstitium as well.

Fibrils could be properly identified and characterized by means of electron microscopy (EM) only, but this procedure and the specific diagnostic expertise is not widely diffused and requires prolonged diagnostic turn-around time; moreover, misleading cases with ambiguous fibrils features can occur as well as early-stage cases with no demonstrable fibrils in the small samples assessed on EM. To overcome these diagnostic issues, in 2018, DNA J homolog subfamily B member 9 (DNAJB9), a heat shock protein involved in the unfolded-protein response, was identified as one of the main constitutive proteins of FGN fibrils deposits, and subsequently proposed as an innovative immunohistochemical (IHC) diagnostic marker of FGN, allowing its distinction from other kidney diseases with structured glomerular deposits [2,7,8].

Our study aims to confirm the diagnostic potential of DNJB9 by evaluating its IHC expression profile in a large monocentric series of FGN enriched with referral cases and including a wide range of differential diagnoses.

## 2. Materials and Methods

### 2.1. Cases Selection Criteria and Database Development

We retrospectively retrieved all cases of FGN and suspected FGN diagnosed at the Pathology Unit of the Città della Salute e della Scienza Hospital in Turin, from January 1992 to June 2022, including external consult cases. Overall, we collected 70 cases corresponding to 77 kidney biopsies and analyzed the related clinical and pathology data to create a database anonymized by a pathology staff member not involved in the study. As a control group, 128 cases of non-FGN glomerular disease (NFGNGD), normal kidney, and additional significant diagnostic differential conditions were retrieved accordingly (Table 1). 

### 2.2. Kidney Biopsy Management and Evaluation

Original slides and ultrastructural images were retrieved and reviewed by three expert nephropathologists to confirm the diagnosis and to assess material adequacy. Regarding the FGN cohort, inclusion criteria were represented by the glomerular deposition of fibrils that (I) were straight, non-branching, and randomly oriented, (II) lacked hollow centers, and (III) resulted positive to immunoglobulin stains on IF.

The collected kidney biopsies were processed and set up for LM, IF, and EM assessments according to our laboratory clinical protocols for routine diagnostics, as previously reported [9,10]. 

Nephropathology routine stains (periodic acid Schiff, Masson’s trichrome, phosphotungstic hematoxylin, and acid fuchsine orange G) and Congo red stain were performed. 

For IF, 4 µm cryostat sections were stained with polyclonal fluorescein isothiocyanate-conjugated antibodies for the detection of IgA, IgG, IgG subtypes (i.e., IgG1, IgG2, IgG3, and IgG4), IgM, C3, C4, C1q, Fibrinogen, and κ and λ light chains. 

Glutaraldehyde-fixed and epoxy resin embedded samples were prepared for EM evaluation. From each sample, 50–100 nm-thick sections were cut and then analyzed using the Philips CM10 (Philips Electronics, Eindhoven, The Netherlands) and the JEM-1400 Flash (Jeol, Peabody, MA, USA) transmission electron microscopes.

### 2.3. DNAJB9 IHC Staining Procedure 

From each kidney biopsy block, two 4 μm thick sections were cut and set up on electrostatic charge slides. DNAJB9 IHC stains were performed using the BenchMark ULTRA automatic immunostainer (Ventana Medical Systems Inc., Tucson, AZ, USA) after slides deparaffinization (EZ Prep Concentrate solution; 10×) and heat-induced epitope retrieval (Ventana Cell Conditioning 1 solution; 32 min). Slides were then stained with the anti-DNAJB9 rabbit polyclonal antibody (PA5-59621; ThermoFisher Scientific, Waltham, MA, USA) using a 1:500 dilution for 32 min at 37 °C. Antigen–antibody reactions were visualized using Ventana OptiView Universal DAB Detection and OptiView Amplification Kits. Counterstaining was performed with Mayer’s Hematoxylin (1 min), followed by differentiation with running water (30 s).

### 2.4. Statistical Analysis

We reported categorical variables as frequencies and continuous variables as mean with standard deviation (SD) or median with intervals. We performed proper tests for continuous (*t*-test or ANOVA test) and categorical (Pearson’s Chi-squared -χ2- test and Fisher’s exact test) variables, using Bonferroni correction for multiple groups correlations. We used the standard *p*-value < 0.05 as the cut-off for the statistical analysis significancy assessment and performed statistical analyses using the Stata 15.0 statistical software (StataCorp, College Station, TX, USA).

## 3. Results

### 3.1. Demographical and Clinical Data of the FGN Cohort

The FGN cohort was composed of 77 kidney biopsies, including 62 internal cases and 15 external consults. Of note, seven patients from the internal cases were biopsied twice at different timepoints (i.e., 14 biopsies). The most represented clinical condition at diagnosis was represented by hypertension (34 cases), followed by autoimmune disease (9 cases). The mean creatininemia resulted in an increase (2.04 mg/dL ± 1.54) as well as a daily mean proteinuria (4.78 g/dL ± 2.78; 38 patients presented nephrotic range proteinuria). Microscopic hematuria was observed in 29 cases, whereas 3 cases presented a monoclonal protein component in the serum (Table 2).

### 3.2. Histopathological and Diagnostic Features of FGN Cases

Among the 77 biopsies of FGN, 4 cases presented an additional concurrent disease, represented by extra-capillary glomerulonephritis ANCA-related with acute tubular necrosis (2 cases), light-chain tubulopathy without crystal formation (1 case), and thrombospondin type I domain-containing 7A (THSD7A)-positive membranous glomerulonephritis (1 case) (Table 3). Additionally, four cases of FGN presented a faint positivity to Congo red stains in terms of tiny, scattered deposits, resulting in a diagnosis of congophilic FGN (Table 3). Of note, six cases were originally diagnosed as early-stage FGN. Finally, four cases did not fully meet the diagnostic criteria of FGN and were originally reported as uncertain for FGN (LM, IF, and EM features could not allow a proper distinction with immunotactoid glomerulopathy).

On LM, the most represented pattern of injury was a mesangial expansion without signs of hypercellularity, that was observed in 49 cases, followed by the membranous-like basement membrane thickening (22 cases) and crescentic (7 cases). Interstitial inflammation and fibrosis were observed in most cases (40 and 52 cases, respectively), the latter mainly with a mild extension (26 cases). The mean number of glomeruli was 18.73 (± 16.38) and the mean percentage of complete sclerotic glomeruli was 28.84 (± 27.32).

**Figure 1:**
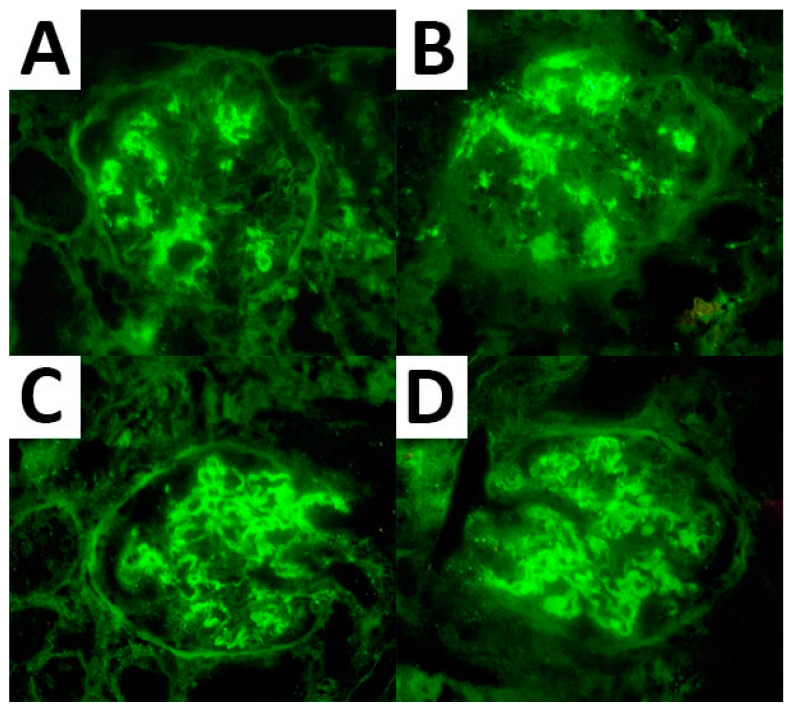
IF expression pattern regarding IgG (**A**), C3 (**B**), and κ (**C**) and λ (**D**) light chain in a case of FGN.

As per the IF analysis, adequate material for expression assessment was available in 67 cases for IgG, 66 cases for C3, and 55 cases for both κ and λ light chains. These markers were expressed in all cases showing a mean expression of 2.6, 2.1, 2.3, and 2.0, respectively. IgG subtyping was available in seven cases and resulted in an IgG1 and IgG4 predominance (seven cases and six cases with a mean expression of 2.3 and 2.4, respectively) (Figure 1). Of note, three cases demonstrated monoclonal light chain deposits (two cases with κ light chain and one case with λ light chain monoclonal deposits).

**Figure 2:**
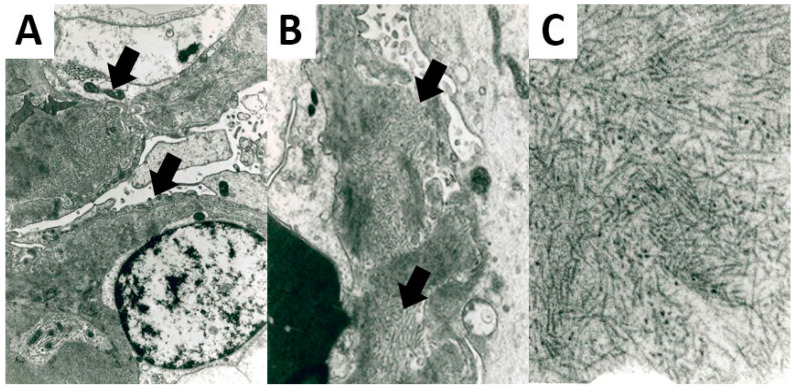
Ultrastructural features of FGN. Fibrils deposits were located in the glomerular basement membrane (**A**) 5200× original magnification; (**B**) 8900× original magnification, showing a straight non-branching structure and a random organization (**C**); 28,500× original magnification.

All FGN cases presented fibrils deposits on the EM analysis. Fibrils showed a straight and non-branching structure, a random organization, and a mean diameter of 20.92 nm (± 3.53) (Figure 2).

Details of the LM, IF, and EM features are reported in Table 4.

### 3.3. DNAJB9 IHC Expression

DNAJB9 stain was retrospectively performed in all FGN cases but resulted evaluable in 74 of the 77 cases of FGN (3 cases did not present glomeruli after sectioning for IHC stain).

DNAJB9 expression was identified in 73 of the 74 available cases diagnosed with FGN, showing mostly a homogenous and strong (so called, “smudgy”) positivity (68 cases), whereas 5 cases presented a moderate and scattered expression in the mesangial axes and occasionally in the glomerular basement membranes. Of note, one of these cases was originally diagnosed as an early-stage FGN. Additionally, four of the DNAJB9-positive cases were originally diagnosed as uncertain for FGN (LM, IF, and EM features could not allow a proper distinction with an immunotactoid glomerulopathy), but presented a strong DNAJB9 expression.

**Figure 3:**
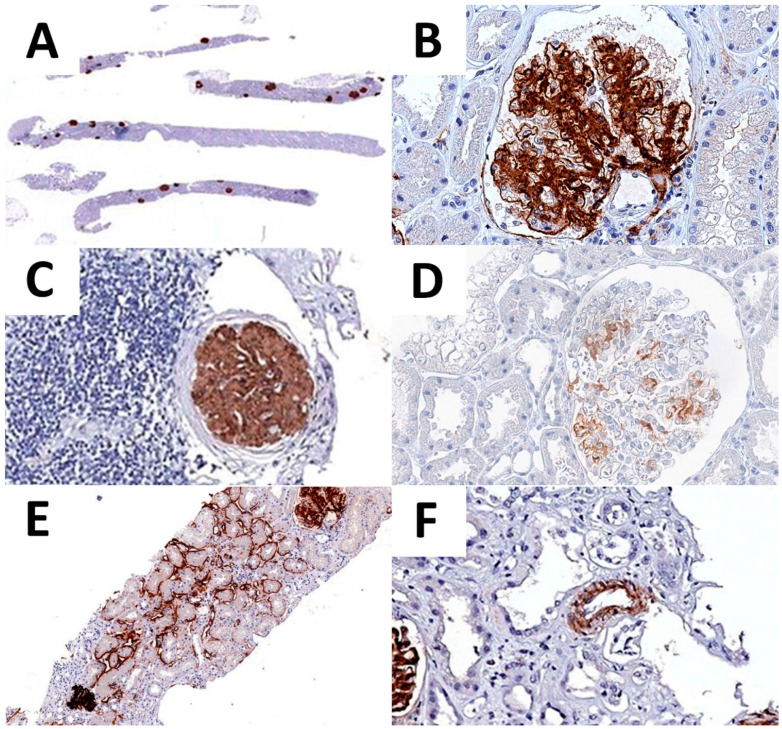
DNAJB9 IHC expression. DNAJB9 glomeruli positivity is recognizable already at low-power magnification (**A**). High-power image of DNAJB9 expression, showing a neat glomerular expression pattern (**B**). DNAJB9 expression is conserved in the glomeruli with intense periglomerular inflammation (**C**). Moderate and scattered DNAJB9 positivity in one of the early-stage FGN cases (**D**). DNAJB9 tubular (**E**) and vascular (**F**) expression.

Regarding the pattern of expression, 43 cases presented an additional extraglomerular DNAJB9 expression. In particular, 35 cases showed a focal positivity of tubular basement membranes, and 7 cases both tubular and vascular (Table 5 and Figure 3).

**Figure 4:**
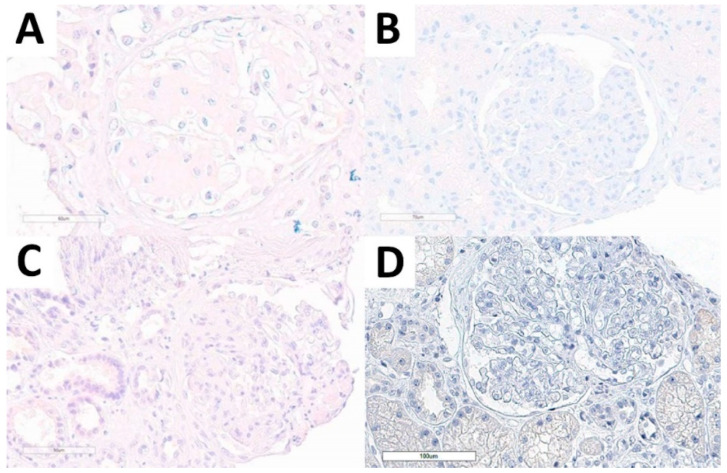
DNAJB9 IHC stain in the non-FGN cohort. DNAJB9-negative stain in amyloidosis (**A**), immunotactoid glomerulopathy (**B**), idiopathic nodular glomerulosclerosis (**C**), and PGNMID (**D**) cases.

Considering the non-FGN cohort, we performed DNAJB9 IHC stain in all cases and all of them were evaluable. None of the cases presented DNAJB9 expression, neither in the kidney diseases with structured glomerular deposits subgroup, nor in normal kidney tissue, or in other relevant diagnostic differential conditions (Figure 4).

The pathologists’ concordance rate in assessing DNAJB9 expression was of 100% in both FGN and non-FGN cohorts, both considering glomerular and extraglomerular expression. DNAJB9 specificity and sensitivity in identifying FGN cases were 100% and 99%, respectively.

### 3.4. Clinicopathological Correlates of DNAJB9 IHC Pattern

An additional aim of our study was to assess the associations between the DNAJB9 expression patterns (e.g., glomerular only vs. glomerular and extra glomerular, moderate vs. strong) with clinical and pathological data. Indeed, we identified a significant correlation between the presence of the endocapillary proliferative histopathological pattern and the DNAJB9 glomerular-only versus the glomerular and extraglomerular pattern of expression (*p* = 0.014) (Supplementary Table S1). However, no other significant correlation emerged, either comparing the intensity of staining (moderate versus strong; Supplementary Table S2) or the concurrent positivity to Congo red staining (congophilic FGN versus non-congophilic FGN; Supplementary Table S3).

## 4. Discussion

Our study confirmed the specificity and sensitivity of DNAJB9 as a diagnostic marker of FGN, regardless of the potential challenges posed by this condition (e.g., early-stage disease, combined conditions, specific rare and atypical variants). Additionally, we identified endocapillary proliferative GN as the only clinicopathological feature associated with the DNAJB9 pattern of expression in terms of glomerular-only compared to glomerular and extraglomerular positivity. Ultimately, our study supports the employment of DNAJB9 IHC staining in the diagnostic routine workup of kidney diseases with structured glomerular deposits.

DNAJB9 was identified being part of the FGN fibrils in 2018 by means of laser microdissection-assisted liquid chromatography–tandem mass spectrometry and subsequently confirmed with IHC analysis [7,8,11]. DNAJB9 was first identified in 2002 as a ubiquitous protein preferentially expressed in endoplasmic reticulum-rich cells (e.g., liver, placenta, and kidneys) [12,13]. As a consequence, DNAJB9 IHC could be positive in normal tissue showing a weak, granular, and focal cytoplasmic positivity [8]. Interestingly, despite its recognition as the most specific component of the FGN fibrils, DNAJB9 transcription did not result upregulate in the kidney tissue of FGN cases, thus suggesting that its involvement in FGN development is secondary to the binding of circulating molecules to misfolded IgG rather than a glomerular “onsite” production [14,15]. Nevertheless, the exact FGN etiopathogenesis is still largely unexplored [2,13,16,17].

Since its first IHC evaluation in 2018, more details are being increasingly revealed regarding the DNAJB9 IHC profile of FGN cases [8,18]. We decided to assess the DNAJB9 IHC profile in our tertiary referral center for nephropathology disease diagnosis to verify its diagnostic potential and identify potential caveats. Our series of FGN largely corresponds to the available literature in terms of clinical data, histopathological (LM, IF, and EM) features, and DNAJB9 IHC profile, but presents few singularities worthy of mentioning. Indeed, in our study we identified four main FGN diagnostic scenarios which could be thoroughly improved by the employment of DNAJB9: (1) differentiating congophilic FGN cases from amyloidosis and, in general, FGN from other glomerular diseases with similar features; (2) identifying early-stage FGN cases; (3) confirming the FGN diagnosis in uncertain or misleading cases; and (4) highlighting the FGN component in combined disease.

One of the main diagnostic needs is to differentiate FGN from other similar conditions, starting with amyloidosis. Indeed, the glomerular involvement in amyloidosis disease is characterized by a potentially overlapping IF (particularly in cases of AHL, AA, and AH amyloidosis with glomerular entrapped Ig) and EM (smaller but similar glomerular fibrils) profiles [1]. Supporting the diagnostic workup, Congo red stain usually allows to accurately differentiate most cases of amyloidosis from FGN. However, there is a small but relevant percentage of FGN cases (up to 4–5%) that showed Congo red positivity, namely congophilic FGN [19,20]. Congophilic FGN could represent a significant diagnostic issue, potentially leading to FGN misdiagnosis with significant consequences on the subsequent patient management [19]. In our series, we identified four congophilic FGN cases that eventually resulted positive to DNAJB9. This data confirmed the diagnostic potential of DNAJB9 in this rare yet misleading scenario, as already observed in other studies [18,19,21]. However, differently from the literature, we did not identify any significant difference regarding fibrils dimension nor any other clinicopathological data comparing congophilic and non-congophilic FGN cases [18,19,21]. Amyloidosis does not represent the only challenging diagnostic differentials when dealing with FGN. Several other conditions can present overlapping features in terms of LM, IF, and EM, such as diabetic nephropathy, immunotactoid glomerulopathy, fibronectin glomerulopathy, cryoglobulinemic glomerulonephritis, and collagenofibrotic glomerulopathy [1,5]. In our study, all these conditions, together with all the other kidney diseases and normal tissues included in our non-FGN control group, resulted negative to DNAJB9, further supporting its specificity in the FGN diagnostic workup.

An additional issue in FGN diagnosis is represented by early-stage disease. Indeed, considering the FGN progressive course eventually leading to end-stage chronic kidney disease and the putative response of early-stage cases to tempestive steroid protocols, its precocious identification is crucial to carefully plan the subsequent clinical management [22]. Unfortunately, initial phases of FGN-mediated glomerular injury are particularly subtle to identify, especially on LM, ultimately delaying the diagnosis. In this setting, cases of early-stage FGN have been reported as DNAJB9-positive in the literature, but described together with other atypical conditions (e.g., late-stage disease, limited glomeruli) [8,18]. We confirm this evidence, as all of the six early-stage cases of FGN of our series expressed DNAJB9. Of note, one of them presented a moderate and focal positivity in the mesangium and glomerular basement membrane, while the others presented strong and smudgy positivity. Interestingly, we also identified this pattern of DNAJB9 positivity (i.e., moderate intensity and focal glomerular expression) in four other cases of our series. In this latter group, the DNAJB9 moderate and focal expression could be explained by the partial loss of antigenicity (the four cases were collected in the mid 1990s), while the early-stage case was collected recently. Therefore, we believe that this finding is of particular interest, as it represents a novel DNAJB9 IHC expression profile in FGN, thus preventing potential misleading interpretations in further series and in the daily diagnostic routine. Of note, we did not identify any specific clinical or histopathological feature related to this pattern of stain.

Cases with ambiguous histopathological features or multiple concurrent diseases in association to FGN are rare, but can occur [3,23]. Our series presented four cases that originally presented ambiguous features using conventional (i.e., without DNAJB9 IHC) diagnostic workup and four cases with concurrent disease. In all these cases, DNAJB9 presented a strong smudgy expression, additionally affirming the usefulness of DNAJB9 staining in assessing FGN.

As far as DNAJB9 is an excellent FGN diagnostic marker, atypical cases do occur, such as DNAJB9-negative FGN or DNAJB9-positive non-FGN cases. To date, few DNAJB9-negative FGN cases were reported in the literature, mostly presenting atypical or unique features [8,18,21]. In particular, Nasr et al. described two DNAJB9-negative FGN cases, both presenting an atypical IF pattern characterized by IgG-only positivity without κ or λ light chains expression. They suggested that in these cases fibrils were probably composed of truncated Ig gamma heavy chain, thus resulting in the atypical staining pattern and absent DNAJB9 expression [8]. Similarly, Andeen et al. identified six DNAJB9-negative FGN cases, all of them showing immune complex-mediated glomerulonephritis and mono-/polytypic fibrillar immune deposits: three cases were subsequently diagnosed differently after rereview (two as probable immunotactoid glomerulonephritis and one as amyloid light-chain (λ)-type amyloidosis with crescentic glomerulonephritis), while the three remaining cases were reported with a descriptive diagnosis compatible with FGN [18]. Finally, Liang et al. described a DNAJB9-negative FGN case characterized by membranous-like nephropathy (LM), monotypic IgG1 and λ light chain expression (IF), and 12–20 nm fibrils (EM) [21]. In our series, we identified a DNAJB9-negative FGN case as well: this 16-year-old male presented a peculiar clinical history characterized by common variable immunodeficiency (CVID), a disease characterized by hypo-/agammaglobulinemia and requiring intravenous monomeric immunoglobulins infusion [24,25]. As the putative etiopathogenetic mechanism of FGN seems to involve the interaction of DNAJB9 with misfolded IgG and C3 fragments, the most accredited hypothesis for DNAJB9-negative FGN cases reported so far involved the deposition of truncated immunoglobulins in the context of a monoclonal gammopathy (so called, heavy chain FGN) [16,26]. However, in our case this condition is not appliable, thus we hypothesized that our patient could have presented somehow an unusual, iatrogenic glomerular deposits of immunoglobulins that did not interact with DNAJB9. To the best of our knowledge, no other cases of FGN developed in the CVID setting has been reported so far. Differently from the literature, we did not identify any cases of non-FGN cases expressing DNAJB9 [8,18].

A comparison between our series data and the major studies addressing DNAJB9 expression in FGN is reported in Table 6.

Ultimately, DNAJB9 could thoroughly reduce the diagnostic turn-around time of FGN. In our institution, we constantly deal with a high throughput of ultrastructural analysis and, therefore, EM characterization requires 1–2 weeks before being fully accomplished, whereas DNAJB9 IHC could be performed in 2 days, allowing expert nephropathologist to easily and quickly confirm the diagnostic suspect of FGN arisen from “conventional” (i.e., LM, IF) histopathological features (particularly, the flocculent IF pattern). In addition, DNAJB9 IHC stain necessitates widely available facilities and no specific training for diagnostic interpretation, whereas EM analysis is restricted to referral centers with dedicated laboratory, technician, and pathologist expertise.

Our study presents some limitations, mainly related to its retrospective nature and the potential biases in the assessment of clinicopathological data due to the extended temporal recruitment; these risks have been countered by the case revision performed by expert nephropathologists before performing the DNAJB9 assessment. We believe that our data enhanced available evidence and provided relevant additional insights.

## 5. Conclusions

Due to the rarity of the disease and the challenging diagnostic workup, FGN still represents an unmet need in kidney pathology with poorly characterized etiopathogenesis, restricted therapeutic approaches, and an overall poor prognosis. However, DNAJB9 demonstrated to represent a specific and sensitive diagnostic marker of FGN, thoroughly improving its identification also in atypical scenarios. Thanks to its routinely implementation, we will hopefully be able to better characterize FGN and eventually enable specific treatments even for early disease.

## Figures and Tables

**Table 1:** Control group diagnoses.

Disease	Cases
Immunotactoid glomerulopathy	22
Membranous glomerulonephritis	16
Amyloidosis	14
Cryoglobulinemic glomerulonephritis	14
Membranoproliferative Glomerulonephritis with Immune Complexes	9
Diabetic Nephropathy	6
PGNMID (Proliferative Glomerulonephritis With Monoclonal IgG Deposits)	5
Proliferative lupus glomerulonephritis	5
Extracapillary proliferative glomerulonephritis ANCA-related	4
Anti-GBM Glomerulonephritis	4
IgA Nephropathy	4
Normal kidney	4
Acute post-infective glomerulonephritis	3
Idiopathic Nodular Glomerulopathy	3
Minimal change disease	3
C3 Glomerulopathy	2
Focal segmental glomerular sclerosis	2
Alport syndrome	1
Cryofibrinogen-associated glomerulonephritis	1
Fibronectin glomerulopathy	1
IgM Nephropathy	1
Light Chain Cast Nephropathy	1
Thin Basement Membrane Disease	1
Kidney Donor	1
Crystal storing histiocytosis	1
Total	128

**Table 2:** Clinical features of the FGN cohort.

Clinical Features	Cases
Male/Female ratio °	37/25
Median age (years) (interval)	56 (16–80)
Clinical condition	
Hypertension	34
Autoimmune diseases *	9
Diabetes	5
HCV	4
Neoplasia ^§^	4
Other ^+^	29
Mean creatininemia (±SD)	2.04 mg/dL (±1.54)
Mean proteinuria (±SD)	4.78 g/dL (±2.78)
Cases with nephrotic range proteinuria (i.e., ≥3.0 g/day)	38
Hematuria	
Micro	29
Macro	1
Protein monoclonal component (serum)	3

°: demographical data of consultants were partial; *: rheumatoid arthritis (3), Basedow disease (2), autoimmune thyroiditis (1), Hashimoto’s Thyroiditis and Autoimmune Gastritis (thyrogastric syndrome) (1); Lupus anticoagulant (LAC) positive (1); systemic lupus erythematosus (1). §: 2 cases of colorectal adenocarcinoma; 1 case of papillary renal cell carcinoma; 1 case of myeloma. +: metabolic syndrome (8), cardiopathy (6), atrial fibrillation (4), stroke (4), cephalea (4), valvulopathy (3), urinary incontinency (3), non-alcoholic liver fatty disease (3), hypercholesterolemia (2), prolonged PTT (2), GERD (2), hyperuricemia (2), deep vein thrombosis (2), HIV (1), MGUS (1)hypothyroidism (1), lumbosciatalgia(1), cervical canal stenosis (1), hemorrhoids sarcoidosis (1), anemia (1), diverticulosis (1), spastic colitis (1), hyperhomocysteinemia (1), arthrosis (1), glaucoma (1), neurosensorial hypoacusis (1), and BPCO (1). IQR: interquartile range; SD: standard deviation.

**Table 3:** FGN cohort characteristics.

FGN	Cases
Combined	4
With light-chain proximal tubulopathy without crystal formation	1
With extra capillary ANCA-related GNF	2
With THSD7A-positive membranous GNF	1
Congophilic	4
Early-stage	6
Uncertain but compatible with FGN	4

GNF: glomerulonephritis; ATN: acute tubular necrosis; THSD7A: thrombospondin type I domain-containing 7A. None of the cases were initially excluded due to material inadequacy/tissue exhaustion.

**Table 4:** Histopathology, immunofluorescence, and ultrastructural characteristics of the FGN cohort.

**Light Microscopy**	**Cases**
Glomerular injury	
Mesangial expansion with no hypercellularity	49
Membranous-like MB-thickening	22
Crescentic	7
Endocapillary proliferative	4
Membranoproliferative	1
Other *	15
Interstitial fibrosis	52
Mild	26
Moderate	19
Severe	7
Interstitial inflammation	40
Mean number of glomeruli (±SD)	18.73 (±16.38)
Mean percentage of sclerotic glomeruli (±SD)	28.84 (±27.32)
**Immunofluorescence**	**Cases (mean intensity)**
IgA	11 (0.3+)
IgG	67 (2.6+)
IgG1	7 (2.3+)
IgG2	2 (1+)
IgG3	2 (0.75+)
IgG4	6 (2.4+)
IgM	22 (0.6+)
C3	66 (2.1+)
C4	23 (0.5+)
C1q	44 (1+)
Fibrinogen	6 (0.31+)
κ light chain	55 (2.3+)
λ light chain	55 (2.0+)
**Electron Microscopy**	
Mean fibrils diameter (±SD)	20.92 nm (±3.53)

*: focal segmental glomerulosclerosis (13), glomerular basement membrane remodeling (2).

**Table 5:** DNAJB9 immunohistochemical expression in the FGN cohort.

Pattern of Expression	Cases
Glomerular	
Negative	1
Moderate and focal	5
Strong/Smudgy	68
No glomeruli	3
Tubular	35
Vascular (arterioles or arteries)	1
Tubular and Vascular	7

**Table 6:** Comparison of studies assessing DNAJB9 in FGN series.

	Our Series	Nasr et al. [8]	Andeen et al. ^+^ [18]	Liang et al. [21]
FGN cases analyzed with DNAJB9	74	84	54	7
positive	73	82	48	6
negative	1	2	6	1
specificity	100%	99.2%	Not clearly specified	Not clearly specified
sensitivity	99%	97.6%	Not clearly specified	Not clearly specified
Congophilic FGN (DNAJB9-positive)	4 (4)	N/A	9 (6 °)	3 (3)
Early-stage FGN(DNAJB9-positive)	6 (6)	Not clearly specified (all of them *)	Not clearly Specified ^§^ (all of them)	N/A
FGN with concurrent kidney disease (DNAJB9-positive)	4 (4)	14 (all of them)	1 (1)	N/A

+: this study mainly focused on atypical FGN cases; °: DNAJB9 was performed in 6 of the 9 available cases; §: early-stage FGN cases were grouped together with other diagnostic challenging cases and eventually reported altogether as positive to DNAJB9; *: early-stage FGN disease in the setting of HCV infection was reported as the putative explanation of the DNAJB9-negative case described in this study.

## Data Availability

The data presented in this study are available on request from the corresponding author. The data are not publicly available due to privacy restriction.

## Supplementary Materials

The following supporting information can be downloaded at: https://www.mdpi.com/article/10.3390/biomedicines10092102/s1, Supplementary Table S1. Clinicopathological correlates of DNAJB9 pattern of expression; Supplementary Table S2. Clinicopathological correlates of DNAJB9 stain intensity; Supplementary Table S3. Clinicopathological correlates of congophilic and non-congophilic FGN cases.

## Abbreviation

ATN	acute tubular necrosis
CVID	common variable immunodeficiency
DNAJB9	DNA J homolog subfamily B member 9
EM	electron microscopy
FGN	fibrillary glomerulonephritis
GBM	glomerular basement membrane
GNF	glomerulonephritis
IF	immunofluorescence
IHC	immunohistochemistry
IQR	interquartile range
LAC	lupus anticoagulant
LM	light microscopy
NFGNGD	non-FGN glomerular disease
PGNMID	proliferative glomerulonephritis with monoclonal IgG deposits
SD	standard deviation
THSD7A	thrombospondin type I domain-containing 7A
