# Supplementary material for: DNAJB9 Is a Reliable Immunohistochemical Marker of Fibrillary Glomerulonephritis: Evaluation of Diagnostic Efficacy in a Large Series of Kidney Biopsies
Source: Biomedicines. 2022 Aug 27;10(9):2102. doi: 10.3390/biomedicines10092102 (PMC9495529; doi:10.3390/biomedicines10092102)
Supplement: Supplementary file 1 [file biomedicines-10-02102-s001.zip › Supplementary Table.pdf]

Features		Glomerular only	Glomerular and Extraglomerular	p-value
**Age**		53.9 (± 13.51)	56.71 (± 12.43)	0.657
**Sex**	**Male**	16	21	0.433
	**Female**	8	17	
**Creatininemia (mg/dL) (± SD)**		1.74 (± 1.08)	2.21 (± 1.74)	0.281
**Proteinuria (g/die) (± SD)**		5.46 (± 3.12)	4.32 (± 2.45)	0.203
**Hypertension**	**Presence**	14	20	0.796
	**Absence**	10	17	
**DM**	**Presence**	1	4	0.640
	**Absence**	23	33	
**Autoimmune disease**	**Presence**	1	8	0.076
	**Absence**	23	29	
**Neoplasia**	**Presence**	1	3	0.544
	**Absence**	23	34	
**HCV**	**Presence**	1	3	0.544
	**Absence**	23	34	
**Hematuria**	**Presence**	14	16	0.159
	**Absence**	6	17	
**Membranoproliferative**	**Presence**	1	0	0.364
	**Absence**	19	35	
**Endocapillary proliferative**	**Presence**	4	0	**0.014**
	**Absence**	16	35	
**Crescentic**	**Presence**	4	3	0.242
	**Absence**	16	32	
**Mesangial expansion with no hypercellularity**	**Presence**	17	32	0.657
	**Absence**	3	3	
**Membranous-like MB-thickening**	**Presence**	8	14	0.775
	**Absence**	12	21	
**Interstitial fibrosis**	**Presence**	21	31	0.877
	**Absence**	3	5	
**Interstitial inflammation**	**Presence**	18	22	0.404
	**Absence**	6	12	
**Fibrils dimension (nm) (± SD)**		20.71 (± 4.07)	21.10 (± 3.09)	0.257

Supplementary Table S1. Clinicopathological correlates of DNAJB9 pattern of expression.

Features		Moderate	Strong	p-value
**Age**		56.33 (± 8.74)	56.28 (± 12.05)	0.595
**Sex**	**Male**	3	33	0.262
	**Female**	0	25	
**Creatininemia (mg/dL) (± SD)**		0.77 (± 0.64)	2.13 (± 1.56)	0.207
**Proteinuria (g/die) (± SD)**		6.73 (± 6.57)	4.63 (± 2.50)	0.205
**Hypertension**	**Presence**	1	33	0.574
	**Absence**	2	24	
**DM**	**Presence**	1	4	0.233
	**Absence**	2	53	
**Autoimmune disease**	**Presence**	0	9	0.455
	**Absence**	3	48	
**Neoplasia**	**Presence**	0	4	0.635
	**Absence**	3	53	
**HCV**	**Presence**	0	4	0.635
	**Absence**	3	53	
**Hematuria**	**Presence**	1	28	0.577
	**Absence**	2	21	
**Membranoproliferative**	**Presence**	0	1	0.890
	**Absence**	1	52	
**Endocapillary proliferative**	**Presence**	1	2	0.056
	**Absence**	0	51	
**Crescentic**	**Presence**	0	7	0.697
	**Absence**	1	46	
**Mesangial expansion with no hypercellularity**	**Presence**	1	47	0.721
	**Absence**	0	6	
**Membranous-like MB-thickening**	**Presence**	0	22	0.403
	**Absence**	1	31	
**Interstitial fibrosis**	**Presence**	1	51	0.692
	**Absence**	0	8	
**Interstitial inflammation**	**Presence**	1	39	0.499
	**Absence**	0	18	
**Fibrils dimension (nm) (± SD)**		22.00 (± 0)	21.00 (± 3.56)	0.784

Supplementary Table S2. Clinicopathological correlates of DNAJB9 stain intensity.

Features		Other FGN	Congophilic FGN	p-value
**Age**		56.79 (± 11.97)	63.00 (± 10.74)	0.812
**Sex**	**Male**	29	1	0.305
	**Female**	20	3	
**Creatininemia (mg/dL) (± SD)**		2.12 (± 1.55)	1.90 (± 0.96)	0.454
**Proteinuria (g/die) (± SD)**		4.42 (± 2.54)	4.45 (± 1.53)	0.319
**Hypertension**	**Presence**	28	2	0.782
	**Absence**	21	2	
**DM**	**Presence**	3	1	0.276
	**Absence**	46	3	
**Autoimmune disease**	**Presence**	8	1	0.536
	**Absence**	41	3	
**Neoplasia**	**Presence**	3	1	0.276
	**Absence**	46	3	
**HCV**	**Presence**	3	1	0.276
	**Absence**	46	3	
**Hematuria**	**Presence**	22	4	0.121
	**Absence**	20	0	
**Endocapillary proliferative**	**Presence**	3	0	0.598
	**Absence**	43	4	
**Crescentic**	**Presence**	7	0	0.400
	**Absence**	39	4	
**Mesangial expansion with no hypercellularity**	**Presence**	40	4	0.441
	**Absence**	6	0	
**Membranous-like MB-thickening**	**Presence**	19	0	0.284
	**Absence**	27	4	
**Interstitial fibrosis**	**Presence**	45	4	0.467
	**Absence**	6	0	
**Interstitial inflammation**	**Presence**	33	4	0.303
	**Absence**	16	0	
**Fibrils dimension (nm) (± SD)**		21.00 (± 3.43)	22.67 (± 5.69)	0.296

Supplementary Table S3. Clinicopathological correlates of congophilic and non-congophilic FGN cases.
